# Production and Anticancer Activity of an L-Asparaginase from *Bacillus licheniformis* Isolated from the Red Sea, Saudi Arabia

**DOI:** 10.1038/s41598-019-40512-x

**Published:** 2019-03-06

**Authors:** S. A. Alrumman, Y. S. Mostafa, Kholood A. Al-izran, M. Y. Alfaifi, T. H. Taha, S. E. Elbehairi

**Affiliations:** 1 Department of Biology, College of Science, King Khalid University, P.O. Box 9004, Abha, 61413 Saudi Arabia; 2 Environmental Biotechnology Department, Genetic Engineering and Biotechnology Research Institute, City of Scientific Research & Technological Applications, P.O. Box: 21934, Alexandria, Egypt

## Abstract

Microbial L-asparaginase (ASNase) is an important anticancer agent that is used extensively worldwide. In this study, 40 bacterial isolates were obtained from the Red Sea of Saudi Arabia and screened for ASNase production using a qualitative rapid plate assay, 28 of which were producing large L-asparagine hydrolysis zones. The ASNase production of the immobilized bacterial cells was more favorable than that of freely suspended cells. A promising isolate, KKU-KH14, was identified by 16S rRNA gene sequencing as *Bacillus licheniformis*. Maximal ASNase production was achieved using an incubation period of 72 h, with an optimum of pH 6.5, an incubation temperature of 37 °C, an agitation rate 250 rpm, and with glucose and (NH_4_)_2_SO_4_ used as the carbon and nitrogen sources, respectively. The glutaminase activity was not detected in the ASNase preparations. The purified ASNase showed a final specific activity of 36.08 U/mg, and the molecular weight was found to be 37 kDa by SDS-PAGE analysis. The maximum activity and stability of the purified enzyme occurred at pH values of 7.5 and 8.5, respectively, with maximum activity at 37 °C and complete thermal stability at 70 °C for 1 h. The *K*_m_ and *V*_max_ values of the purified enzyme were 0.049995 M and of 45.45 μmol/ml/min, respectively. The anticancer activity of the purified ASNase showed significant toxic activity toward HepG-2 cells (IC_50_ 11.66 µg/mL), which was greater than that observed against MCF-7 (IC_50_ 14.55 µg/mL) and HCT-116 cells (IC_50_ 17.02 µg/mL). The results demonstrated that the Red Sea is a promising biological reservoir, as shown by the isolation of *B. licheniformis*, which produces a glutaminase free ASNase and may be a potential candidate for further pharmaceutical use as an anticancer drug.

## Introduction

ASNase (EC 3.5.1.1) is an important enzyme used in the pharmaceutical, biosensor and food industries^1^ and has anticarcinogenic potential for the treatment of acute lymphoblastic leukemia, lymphomas and other cancers^2,3^. ASNase selectively targets the metabolism of cancer cells by exploiting deficiencies in metabolic pathways and catalyzing the degradation of L-asparagine into L-aspartic acid and ammonia, causing nutrient starvation of cancer cells and bringing about their demise^4^. Thus, there is a need for novel and robust ASNases from new microorganisms that exhibit improved stability, lower glutaminase activity, high substrate affinity, and low Km values for use as therapeutics. Two types of commercial ASNase are currently in clinical use for chemotherapy, enzymes from *Erwinia chrysanthemi* and *E. coli*. However, these enzymes have drawbacks in that they exhibit low substrate specificity and high glutaminase activity^5^, the latter of which can cause liver dysfunction, pancreatitis, leucopenia, neurological seizures, and coagulation abnormalities that can lead to intracranial thrombosis or hemorrhages. Therefore, it is important to identify sources and methods of producing greater amounts of glutaminase free ASNases and exhibit high substrate affinity and therapeutic activity^6,7^. A wide range of microbes have been shown to be valuable sources of enzymes, such as *Pichia pastoris*, *Saccharomyces cerevisiae*, *E. coli*, *Aerobacter*, *Pseudomonas, Bacillus*, *Xanthomonas*, *Serratia*, and *Streptomyces*^8^. Recently, ASNases from *E. coli* and *Erwinia carotovora* produced by submerged fermentation were approved for use in medical applications by the United States Food and Drug Administration^9,10^. The Red Sea of the Saudi Arabia has yet to be thoroughly investigated with respect to its harboring microbes with biopharmaceutical and biotechnological potential^11^. Marine microbe-derived ASNases may be more effective and have fewer side effects as therapeutic agents for acute lymphoblastic leukemia treatment than traditional sources of these enzymes^12^. The enhanced production of novel glutaminase free ASNase from *Pectobacterium carotovorum*^13^ and *Erwinia carotovora*^14^ was achieved by submerged fermentation in batch and fed batch bioreactors. Cell immobilization is considered to be a promising approach for enhancing fermentation processes for ASNase production^15^. The benefits of using immobilized cells include the facilitation of continuous operations over an extended period of time, potential recycling of immobilized beads and a simpler means of harvesting these products, reactor productivity, higher catalysis efficiency and the development of economical methods, as well as lowering the cost of industrial processes^7^. The purification of ASNases is important to promote their characterization and therapeutic use with fewer adverse effects^16^. Thus, the goal of the present study was to isolate and screen marine bacteria for the production of glutaminase free ASNases from the Red Sea, Saudi Arabia. The optimization of enzyme production using free and immobilized cells and an evaluation of its properties and potential as an anticancer agent against different human cancer cell lines was also investigated.

## Materials and Methods

### Chemicals

The chemicals used for enzyme production and purification were purchased from Sigma-Aldrich Chemical Co., USA. Nessler’s reagent was purchased from Fluka (Buchs, Switzerland). The chemicals and markers used for the molecular identification of ASNases were obtained from Bio-Rad Laboratories (USA).

### Sample collection

Between January and March 2017, water samples were collected from the Red Sea off the southwestern coast of Saudi Arabia from three stations: The Al-Shuqaiq coast (41° 59′22.5″E, 17° 42′35.0″N), the Al-Haridah coast (41° 55′17.8″E, 17° 45′58.5″N) and the Al-Birk coast (41° 31′40.5″E, 18° 13′49.4″N). The water samples were collected in 500-mL glass screw cap bottles and stored at 4 °C until processed.

### Isolation of marine bacterial strains

Two methods were used to isolate marine bacteria: (1) the water samples (100 ml) were filtered through a Millipore membrane filter (0.22 µm), which was subsequently aseptically transferred onto agar-solidified medium; and (2) the water samples were serially diluted, and 100 µl of the 10^−1^,10^−2^, 10^−3^ and 10^−4^ dilutions were spread onto agar-solidified medium. All of the inoculated plates were incubated at 37 °C for 3 days. The composition of the asparagine agar^17^ was as follows (w/v): 0.6% beef extract, 1% peptone, 0.33% KH_3_PO_4_, 0.1% L-asparagine and 1.5% agar, pH 6.5.

### Screening of bacterial isolates for ASNase production

The bacterial isolates were screened for ASNase production using the rapid plate assay method^18^, which was performed using asparagine agar medium containing 0.3 ml of 2.5% phenol red. After incubating at 37 °C for two to three days, the pink zone around the colonies was measured, and an enzyme index was calculated using the equation: Enzyme index = Pink zone (mm)/Colony diameter (mm).

### Identification of promising isolates via 16S rRNA gene sequence analysis

Bacterial genomic DNA from the promising isolates was extracted from 5-ml cultures grown overnight in nutrient broth using a modified QIAamp DNA Mini kit (Qiagen Inc., Valencia, CA)^19^. The extracted DNA from each bacterial isolate was used as a template for amplification of the 16S rRNA gene using the universal primers 5′ CCA GCA GCC GCG GTA ATA CG 3′ and 5′ ATC GG(C/T) TAC CTT GTT ACG ACT TC 3′. The 50-µL PCR mixtures contained the following components: 10 mM Tris-HCl (pH 8.3), 50 mM KCl, 2.5 mM MgCl_2_, each dNTP at 0.2 mM, 1.25 IU of Taq polymerase, each primer at 0.2 µM, and 1 µL of DNA template, with the final volume brought up to 50 µL with water. PCR was performed using the following thermocycling program: 10 min of denaturation at 94 °C, followed by 35 cycles of 1 min denaturation at 94 °C, 1 min annealing at 55 °C, a 2 min extension at 72 °C, and a final extension for 10 min at 72 °C. Subsequently, 5 µL of the PCR products were analyzed on a 1.5% agarose gel made in 0.5× of Tris/Borate/EDTA (TBE) buffer and containing ethidium bromide. An electrophoresis unit was used to run the gel for 30 min at 150 V. The migrated bands were observed under UV light and photographed using a gel documentation system. To verify the presence of appropriately sized amplicons, the PCR products for each isolate were compared with a 1 kb DNA ladder. Products of the correct size were purified using a TaKaRa Agarose Gel DNA purification kit Ver. 2.0 and were sequenced in both directions using an ABI 3730 automated sequencer (Macrogen, Korea). The obtained sequences for the selected isolates were aligned and compared with the sequences deposited in GenBank (http://blast.ncbi.nlm.nih.gov/Blast.cgi). To determine the taxonomic position of the isolates, a phylogenetic tree was constructed with MEGA version 5.0 using a neighbor-joining algorithm. The Jukes-Cantor distance estimation method with bootstrap analyses for 1000 replicates was also performed. The nucleotide sequences of the amplified 16S rRNA genes of the strains reported in this study have been deposited in the GenBank nucleotide sequence database under the accession number MG580926.

### Immobilization of ***B. licheniform****is* by entrapment in Ca-alginate

The freely suspended *B. licheniformis* cell inoculum was cultured in asparagine broth medium at 37 °C and 250 rpm for 24 h. To prepare the immobilized cell inoculum^10^, the wet cells were prepared after centrifugation (10,000 × *g* for 20 min) of the freely suspended cell inoculum and mixed with 50 ml of 3% (w/v) sodium alginate solution, after which the inoculum was stirred for 10 min to make the mixture uniform. The obtained mixture was extruded dropwise through a 5-ml pipette into a 1.5% (w/v) CaCl_2_ solution. The bacterial cells were entrapped when the alginate drops solidified and formed capsules upon coming into contact with the CaCl_2_ solution. Subsequently, the capsules were allowed to harden for 1.0 hour, after which they were washed with a sterile saline solution (0.09% NaCl) and preserved in CaCl_2_ solution in the refrigerator at 4 °C. All operations were performed aseptically under a laminar flow unit.

### Scanning electron microscope (SEM)

Surface characterization and microscopic analysis of gold coated free bacterial cells and immobilized calcium alginate capsules were carried out using energy-dispersive analysis in a SEM (Jeol Jsm 6360 LA, Japan).

### ASNase production

Erlenmeyer flasks (250 ml) containing 50 ml of asparagine broth medium were inoculated with 4 g of the alginate gel. For the freely suspended cell cultures, the asparagine agar medium was inoculated with bacterial cells equivalent to those used in the immobilized cultures. Fermentation was performed with the free and immobilized cells at 37 °C for three days at 250 rpm. Subsequently, the cultures were centrifuged (10,000 × *g* for 20 min) and the supernatants were used as the enzyme source. The cell growth in the freely suspended cultures and that of the cells immobilized in the alginate matrix was determined as cell dry weight. The total weight of the bacterial cell-containing alginate gel capsules was compared with the alginate capsules without cells to determine the cell biomass. Several factors affecting ASNase production were optimized by varying one factor at a time, namely, incubation period (12–96 h), pH (5.0–8.0), temperature (25–50 °C), agitation rate (0.0–300 rpm) carbon sources (glycerol, fructose, glucose, maltose, sucrose, dextrin and starch), and nitrogen sources (asparagine, yeast extract, urea, casein, ammonium sulfate, ammonium chloride, sodium nitrate and potassium nitrate).

### Determination of ASNase and glutaminase activity

A reaction mixture containing 0.5 mL of enzyme, 0.5 mL of 0.05 M Tris-HCl buffer (pH 7.4), 0.5 mL of 0.04 M asparagine (L-glutamine was used as the substrate to assess glutaminase activity) and 0.5 mL of distilled water to bring the final volume to 2.0 mL was incubated for 30 min. The reaction was stopped by adding 0.5 mL of 1.5 M trichloroacetic acid, and the mixture was then centrifuged at 4025 × *g* for 10 min. The absorbance of a mixture containing 0.1 mL of the supernatant, 3.7 mL of distilled water and 0.2 mL of Nessler’s reagent was measured using a spectrophotometer at 500 nm^20^. One unit of ASNase activity was reported as the amount of enzyme that liberated 1 µmol of ammonia per ml per min.

### Protein determination

The method of Lowry *et al*.^21^ was used to establish the enzyme concentration. A stock solution of standard protein, bovine serum albumin at a concentration of 1000 μg/mL, was prepared. Folin-Ciocalteu reagent was added to each sample, and after incubating for 30 min, the absorbance was measured at 660 nm.

### Purification of ASNase

Ammonium sulfate was added to the cold crude enzyme to reach a saturation of 70%. The mixture was left overnight at 4 °C and then was centrifuged at 4025 × g for 20 min. The precipitate obtained was dissolved in the minimum volume of 50 mM Tris-HCl buffer (pH 7.4) and was dialyzed against the same buffer overnight at 4 °C to remove the salts. The dialyzed fraction was loaded onto a Sephadex G-100 column (45 × 1.5 cm) that was pre-equilibrated with 0.05 M Tris-HCl buffer (pH 8.6). The protein was eluted with 0.05 M Tris-HCl buffer (pH 7.4) containing 0.1 M KCl. The fractions were collected and assayed for protein and enzyme activity^22^. The fraction with the highest level of enzyme activity was lyophilized, and the resulting powder was stored at 4 °C.

### Sodium dodecyl sulfate polyacrylamide gel electrophoresis (SDS-PAGE)

To generate whole cell lysates, cells were resuspended in 200 µL of lysis buffer (62.5 mM Tris/HCl, pH 6.8) (Mllinckrocit, USA), 2% sodium dodecyl sulfate (SDS), 15% glycerol, 5% 2-mercaptoethanol and 0.001% bromophenol blue (tracking dye). The whole cell lysates were boiled for 5 min at 100 °C and then were loaded onto a 15% SDS polyacrylamide gel and run at 100 V. The separated proteins were stained with Coomassie blue. Subsequently, the gel was transferred to de-staining solution to remove unbound dye from the gel and leave the stained proteins as visible blue bands, after which the gel was imaged using a Gel Documentation System.

### Kinetic properties of the purified ASNase

The optimum pH for ASNase activity was determined over a pH range of 5.0 to 10.0. For pH stability studies, enzyme preparations at various pH values were incubated for 1 h, and then, the relative activity was determined under the standard assay conditions. The optimum temperature for enzyme activity was determined by assaying ASNase activity at different temperatures ranging from 30 to 100 °C. In addition, the thermal stability of the enzyme was determined by incubating the lyophilized enzyme at the same temperatures for 1 h and assaying ASNase activity^3^.The kinetic parameters for ASNase (*K*_m_ and *V*_max_) were determined using L-asparagine as a substrate at concentrations ranging from 0.0 to 0.1 M. The Michaelis-Menten parameters were determined from Lineaweaver-Burk plots using the equation derived from linear-regression analysis of the curve^3^.

### Anticancer assay

The cytotoxicity assay^23^ was performed using human cancer cell lines) MCF-7 breast, HCT-116 colon and HepG2 live) obtained from Vacsera (Egypt). The cells were maintained in Roswell Park Memorial Institute (RPMI) media supplemented with 100 µg/mL streptomycin, 100 U/mL penicillin and 10% heat-inactivated fetal bovine serum in a humidified atmosphere containing 5% CO_2_ (v/v) at 37 °C. In addition, the cells were sub cultured twice a week. The cytotoxicity of the enzyme was tested against selected cancer cell lines using the sulforhodamine B (SRB) assay. Exponentially growing cells were collected using 0.25% trypsin-EDTA and plated in 96-well plates at 2000 or 100 μL/well. The cells were exposed to different concentrations of ASNase (0.0–100 μg/mL) for 72 h and were subsequently fixed with 100 μL/well of trichloroacetic acid (10%) for 1 h at 4 °C. After extensive washing, the cells were exposed to a 0.4% SRB solution for 10 min in a dark place and then were washed with 1% glacial acetic acid to remove unbound dye. After drying overnight, the SRB dye was solubilized with 50 μL/well of 10 mM Tris-HCl (pH 7.4) for 5 min on a shaker at 1600 rpm. The optical density (OD) of each well was measured at 570 nm using a microplate reader. Control wells contained only cells and medium without treatment, and the positive control was doxorubicin. The IC_50_ values were calculated using sigmoidal concentration response curve fitting models that were generated with Sigma Plot.

### Statistical analysis

Analysis of variance using one-way ANOVA was performed, and all significant differences at *P* ≤ *0.05* were determined using Minitab for Windows version 15. The error bars represent the standard error of the mean for n = 3. Sigma Plot was used to calculate the IC_50_ values.

### Compliance with ethical standards

This article does not contain any studies with human participants or animals performed by any of the authors.

## Results

### Isolation and screening of marine bacteria producing ASNase from the Red Sea

Forty marine bacterial isolates were isolated from the three marine coastal sites via the Millipore membrane filter method while, serial dilution method was not feasible. The greatest number of bacteria were isolated from the Al-Haridah coast (15 isolates), followed by the Al-Birk coast (14 isolates) and the Al-Shuqaiq coast (11 isolates). All bacterial isolates were screened for their ability to produce ASNase using a qualitative rapid plate assay based on their ability to form a pink zone around colonies on asparagine agar plates containing a phenol red indicator. Twenty-eight isolates showed intense pink zones around the colonies. Four bacterial isolates exhibited a notable ability to produce ASNase as assessed by the diameters of the halos, including KKU-KH14 (1.75 mm) and KKU-KH17 (2.37 mm) from the Al-Haridah coast, KKU-KSh6 (2.0 mm) from the Al-Shuqaiq coast and KKU-KB 27 (1.7 mm) from the AL-Birk coast. By evaluating the morphological properties of the colonies displaying ASNase production potential through the plate assay, 10 isolates were selected for the subsequent in-depth studies.

### ASNase production by free and immobilized cells of 10 promising bacterial isolates

**Figure 1:**
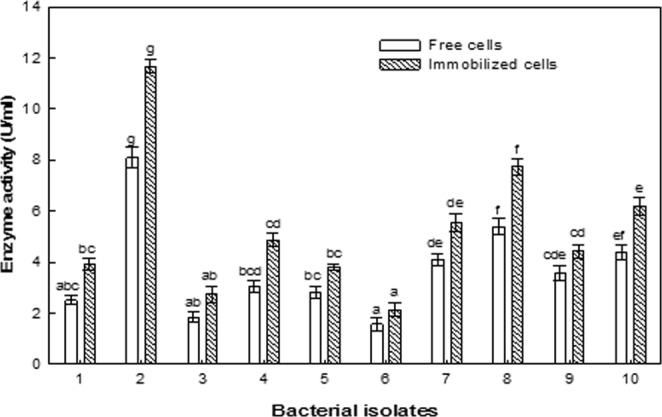
ASNase production by freely suspended and immobilized cells of promising bacterial isolates. 1: KKU-KH12, 2: KKU-KH14, 3: KKU-KH16, 4: KKU-KH17, 5: KKU-KH18, 6: KKU-KH20, 7: KKU-KH 38, 8: KKU-KB27, 9: KKU-KB36, 10: KKU-KSh6. The values in the same column followed by the letter (S) were not significantly different at *P* ≤ 0.05.

**Figure 2:**
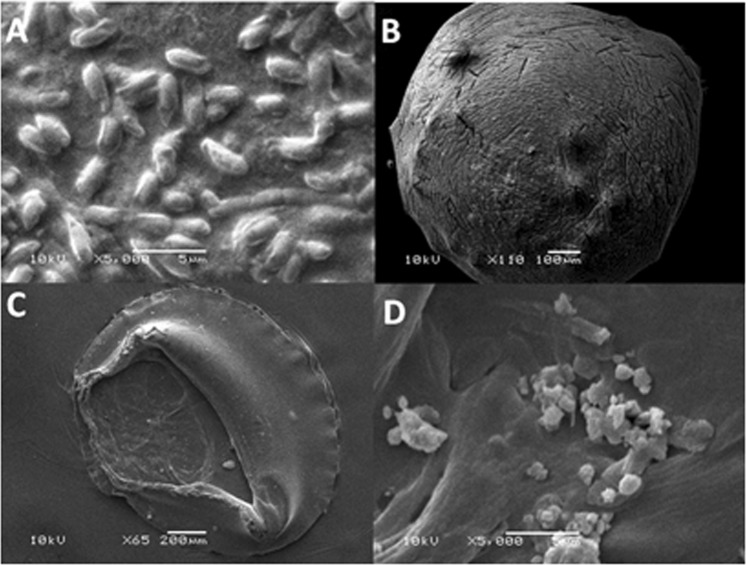
Scanning electron micrograph showing (**A**) free *B. licheniformis* cells (**B**) a capsule of immobilized bacterial cells in alginate polymeric material, (**C**) a semi-sphere capsule and (**D**) bacterial cells inside the cavities of the capsule.

The bacterial isolates with promising ASNase activity were subjected to further screening for the production of ASNase through submerged fermentation in a shaking incubator using two inoculum methods, freely suspended and alginate-immobilized bacterial cells. ASNase production was measured via spectrophotometry using Nessler’s reagent method. The results (Fig. 1**)** indicate that the immobilized cells exhibited greater enzyme production than the free cells. The amount of enzyme produced by free bacterial cells ranged from 1.56 to 8.1 U/mL, whereas that produced by immobilized cells ranged from 2.13 to 11.66 U/mL. The highest enzyme production was achieved by the bacterial isolate KKU-KH14 obtained from the Al-Haridah coast, producing 8.1 U/mL using the freely suspended cell inoculum and 11.66 U/mL using the immobilized cell technique. The morphological appearance of the free and immobilized bacterial cells in calcium alginate capsules were investigated using high power magnifications of a scanning electron microscope (SEM). The photographed free cells showed the bacilli appearance of the *B. licheniformis* strain (Fig. 2A). Additionally, no other bacterial morphologies were detected, which indicates the purity of the used bacterial sample. On the other hand, the spherical shape of the alginate capsules was a clear sign for the proper polymerization of the alginate polymer using calcium ions as a cross linker. Moreover, the proper gold coating of the capsules was under a condition of complete dryness of the scanned capsules, which in turn kept the bacterial cells inside the alginate capsules (Fig. 2B). The investigation of the bacterial cells inside the polymeric capsule was detected using a semi sphere of the prepared capsule (Fig. 2C). The bacterial cells were detected inside the polymeric semi sphere with slightly different shape than seen for free cells. The difference in the bacterial bacilli shape may be attributed to the complete coverage of the cells with the viscous polymeric material that transformed the bacterial shape into slight spherical shape (Fig. 2D). These results indicated the proper immobilization of the bacterial cells inside the polymeric capsules, in addition to the predicted prolonged shelf life of the dried immobilized capsules which have the ability to allow the bacterial cells to restore their activity after exposure to humidity conditions.

### Identification of strains via 16S rRNA gene sequencing and phylogenetic analysis

**Figure 3:**
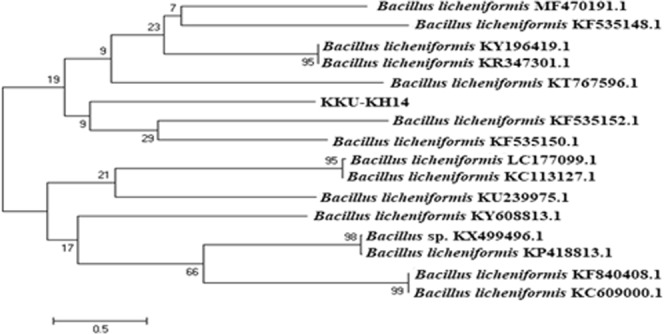
Phylogenetic relationship between the KKU-KH14 bacterial isolate and other 16S rRNA gene sequences of published strains.

To identify and determine the correct phylogenetic position of the selected promising isolate, KKU-KH14, molecular genetic identification was performed. First, the genomic DNA was extracted from the isolated bacterial strain using a modified QIAamp DNA Mini kit. Amplification and sequencing of the 16S rRNA gene fragment was performed by Macrogen, Korea, using the universal primers 27F and 1492R. The obtained sequence data of the16S rRNA gene was compared with the sequences of 16S rRNA regions in GenBank using a BLAST search of the National Center for Biotechnology Information (NCBI) databases (https://blast.ncbi.nlm.nih.gov/Blast.cgi?PROGRAM=blastn&PAGE_TYPE=BlastSearch&LINK-LOC=blasthome). An alignment of the 16S rRNA gene sequence of the isolate, along with sequences obtained from the BLAST search and MACROGEN results, revealed that the strain KKU-KH14 exhibited 99% similarity to *B. licheniformis*. Various sequences taken from the GenBank database were used to build the phylogenetic tree to determine the phylogenetic position of the strain (Fig. 3) and the accession number of bacterial strain sequence information is MG580926.

### Optimization of ASNase production

The optimization of ASNase production by immobilized cells of *B. licheniformis* was investigated using submerged fermentation in an incubator shaker. The cells were cultured in asparagine medium with an initial pH of 6.5 at 37 °C and 250 rpm.

### Effect of time course

**Figure 4:**
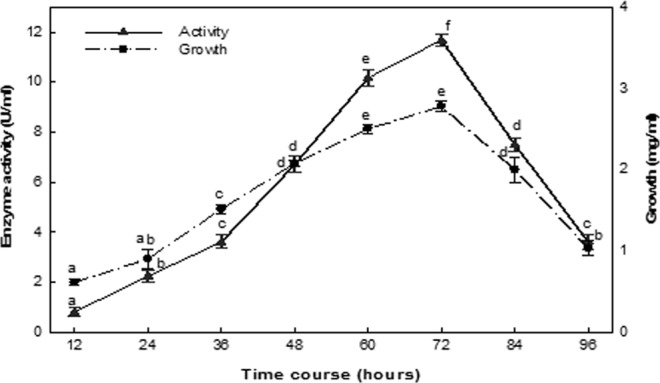
Effect of time course. The values indicated with the same letter (s) on the same line were not significantly different at *P* ≤ 0.05.

The results indicated that the growth of *B. licheniformis* cells entrapped in a calcium alginate matrix increased gradually over a period of up to 72 h of culturing at the stationary growth phase (Fig. 4). After 24 h of incubation, the production was only 19.10% of the maximal observed value, gradually increasing up to 36 h (30.84%), with exponential increase was observed at 72 h. Additional culturing after the optimum culture period did not increase enzyme production but rather strongly decrease in enzyme production and bacterial growth were observed.

### Effect of initial pH

**Figure 5:**
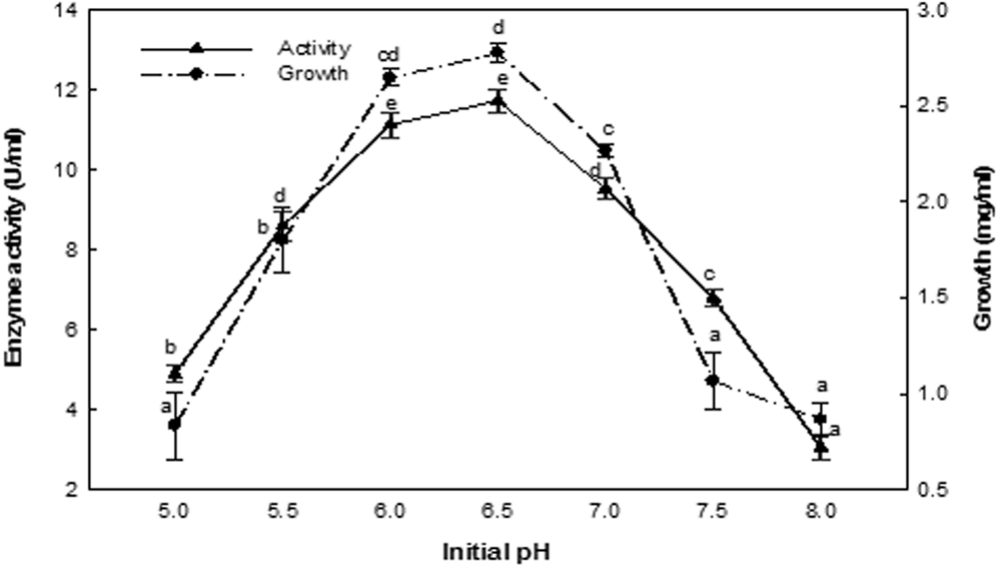
Effect of the initial pH. The values indicated with the same letter (s) on the same line were not significantly different at *P* ≤ 0.05.

The enzyme production was altered according to the initial pH of the medium. The enzyme production increased up to pH 6.5, which was the most favorable value for ASNase production and bacterial growth (Fig. 5). However, beyond this pH value, the production markedly decreased. The ASNase production at pH 6.0 was comparable to that observed at pH 6.5, as the yield decreased by only 5.11%, while at pH 7.5 the yield decreased by 18.77%.

### Effect of incubation temperature

**Figure 6:**
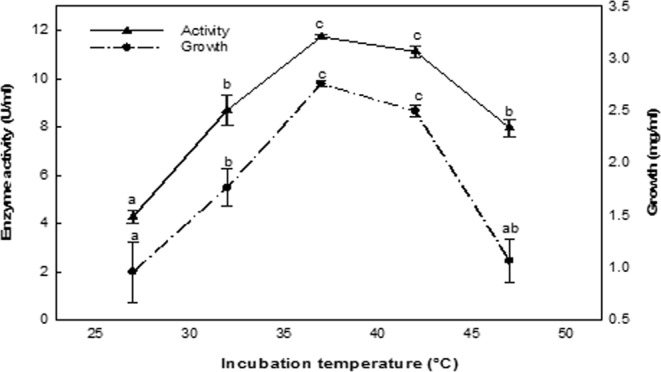
Effect of the incubation temperature. The values indicated with the same letter (s) on the same line were not significantly different at *P* ≤ 0.05.

The results demonstrated a significant relationship between ASNase production and the incubation temperature up to 37 °C, where an ASNase yield of 11.74 U/ml was observed (Fig. 6). A significant reduction in enzyme production was observed compared to the optimum temperature value, decreasing by 5.19 and 32.19% at 42 and 47 °C, respectively.

### Effect of agitation rate

**Figure 7:**
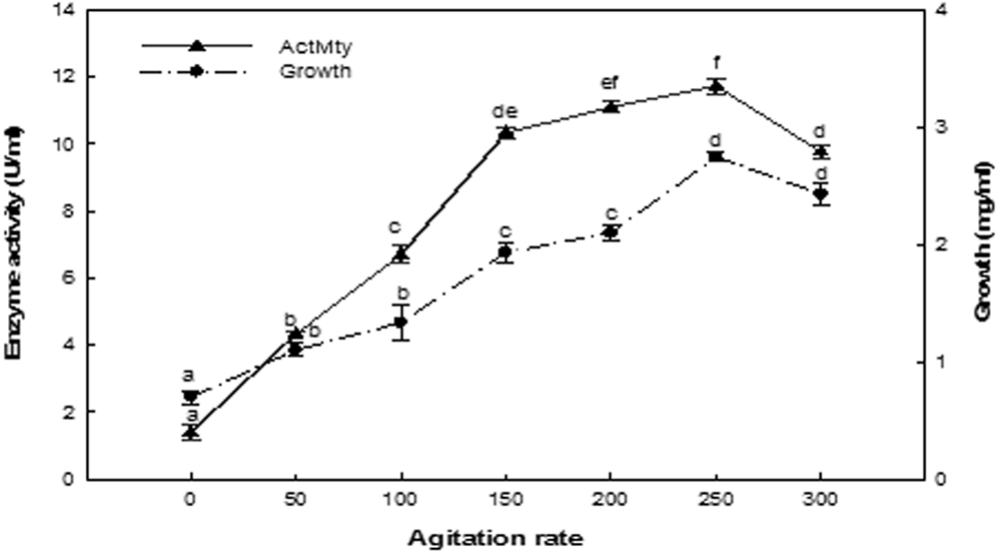
Effect of the agitation rate. The values indicated with the same letter (s) on the same line were not significantly different at *P* ≤ 0.05.

The maximal enzyme production was increased by agitation, with enzyme yield significantly increasing with shaking at 250 rpm (Fig. 7). Under static conditions, enzyme production decreased, reaching 11.93% of the maximal observed value. Furthermore, increasing the agitation rate above the optimum value was unfavorable for ASNase production.

### Effect of carbon sources

**Figure 8:**
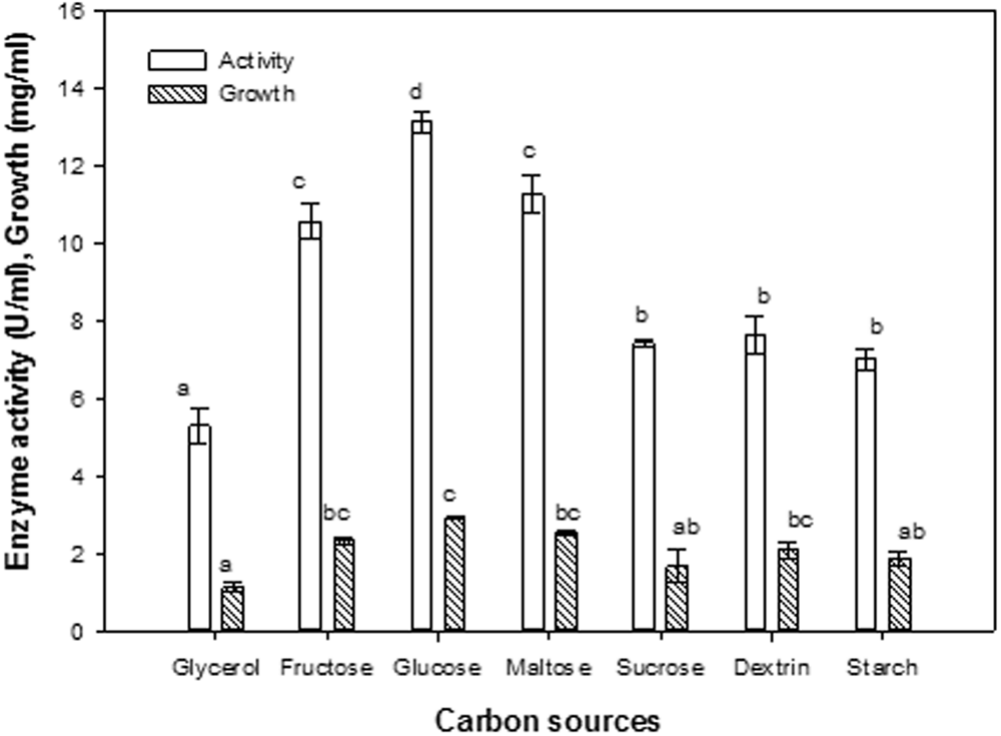
Effect of carbon sources. The values indicated with the same letter (s) in the same column were not significantly different at *P* ≤ 0.05.

Experiments were performed to assess the ability of the *B. licheniformis* isolate to utilize different carbon sources (glycerol, fructose, glucose, maltose, sucrose, dextrin and starch) for the production of ASNase. The carbon source used had a clear effect on ASNase production, with glucose being the most preferred carbon source for enzyme production, reaching 11.2% more than the maximal observed value (Fig. 8). Interestingly, the other tested carbon sources did not facilitate bacterial growth and enzyme production, with the lowest production of ASNase observed using glycerol and starch as carbon sources, reaching 40.36 and 53.31% less than the maximal observed value, respectively.

### Effect of nitrogen sources

**Figure 9:**
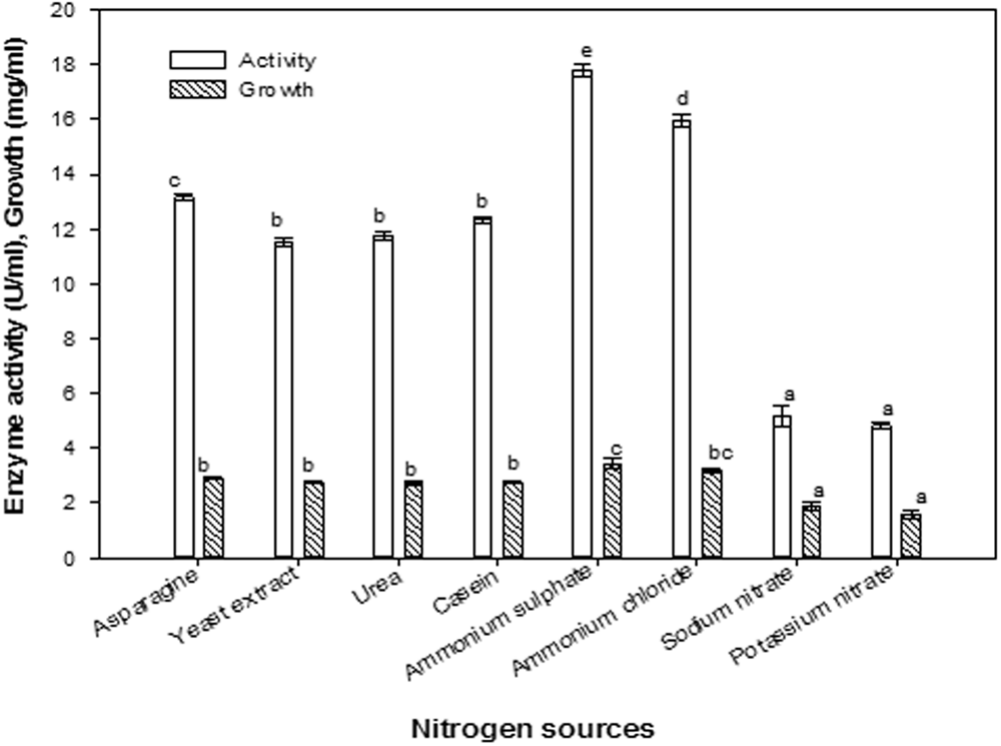
Effect of nitrogen sources. The values indicated with the same letter (s) in the same column were not significantly different at *P* ≤ 0.05.

The effect of different nitrogen sources (asparagine, yeast extract, urea, casein, ammonium sulfate, ammonium chloride, sodium nitrate and potassium nitrate) on ASNase production was investigated. The results showed that ammonium salts were the best source of nitrogen with respect to ASNase yield (Fig. 9) Ammonium sulfate was observed to be the optimum source of nitrogen for *B. licheniformis*, resulting in 35.56% increase in enzyme production. The yeast extract, urea and casein did not show any significant changes in enzyme production, whereas nitrate salts, sodium nitrate and potassium nitrate significantly reduced enzyme production between 71.01 and 72.86% of the maximal observed value.

### Purification of ASNase

ASNase extracted from *B. licheniformis* was purified by ammonium sulfate precipitation, dialysis and gel filtration chromatography using a Sephadex G-100 column (Table 1). In the twelve fractions obtained from the cell free extract, one fraction exhibited ASNase activity. The specific activity of the enzyme and the purity increased with every step of purification, whereas the total protein, total activity and yield decreased proportionally. For the crude enzyme, a yield of 114.4 mg of protein and 890 U of ASNase activity were obtained, with a specific enzyme activity approximately 7.78 U/mg. The purification of the crude enzyme yielded 11.5 mg of protein and 415 U of enzyme activity, with an increased specific activity of 36.08 U/mg. Furthermore, a 4.63-fold purification and a 46.62% yield of the enzyme were obtained.

**Table 1:** Summary of purification procedure of ASNase.

Purification Steps	Enzyme activity (U)	Protein (mg)	Specific activity (U/mg)	Fold Purification	Yield (%)
Crude extract	890.0	114.4	7.78	1.00	100
Ammonium sulfate and dialysis	790.5	83.55	9.46	1.21	88.82
Gel filtration chromatography	415.0	11.50	36.08	4.63	46.62

**Figure 10:**
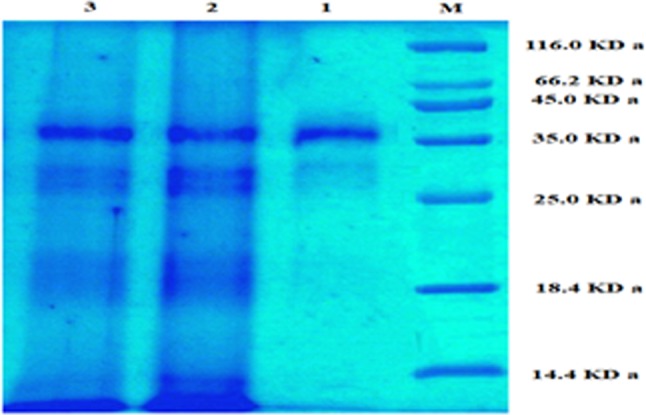
SDS-polyacrylamide gel electrophoresis of the purified ASNase from *B. licheniformis*. M: Protein marker; 1: Sephadex G-100-purified ASNase; 2: Ammonium sulfate-precipitated ASNase; 3: Cell free crude ASNase preparation.

The homogeneity and the molecular weight of the purified ASNase was determined by sodium dodecyl sulfate-polyacrylamide gel electrophoresis (SDS-PAGE). The results demonstrated the purity of the ASNase preparation, as only a single distinctive protein band was observed with an apparent molecular weight of 37 kDa when compared with the standard molecular weight markers (Fig. 10). The SDS-PAGE and glutaminase activity assay results of the ASNase protein demonstrated the success of the enzyme purification and showed that the purified enzyme exhibited no glutaminase activity.

### Kinetic properties of the purified ASNase

**Figure 11:**
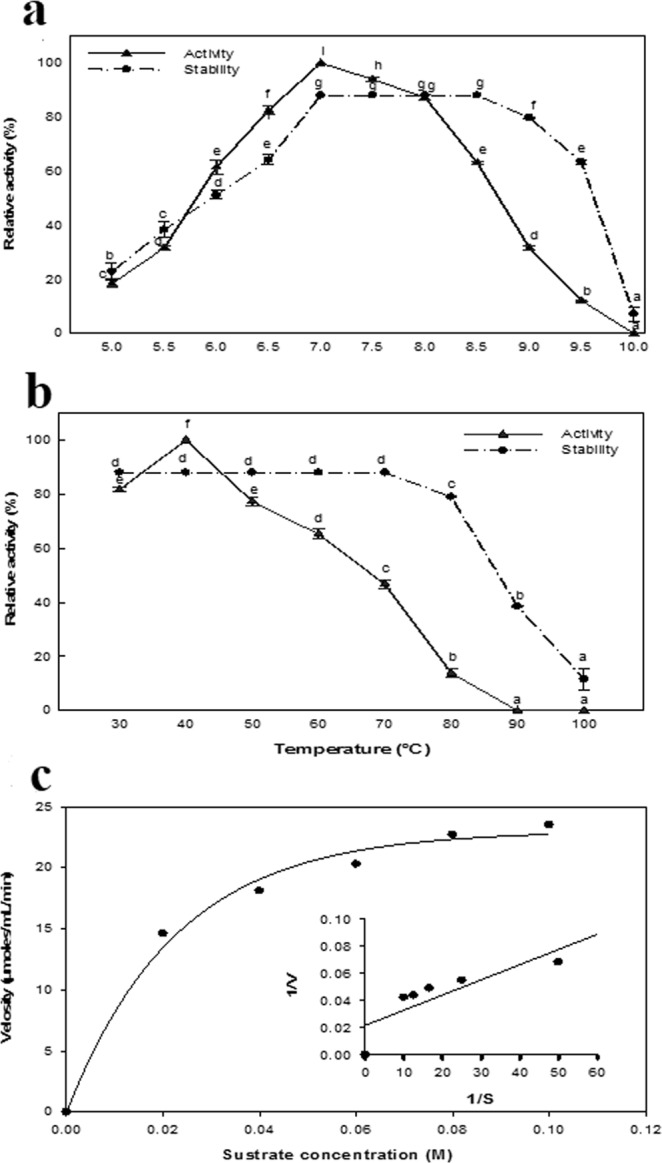
Effect of pH (**a**) and temperature (**b**) on the activity and stability of ASNase. The values indicated with the same letter (s) were not significantly different at *P* ≤ 0.05. The plot of the reaction velocities (V) vs. substrate concentration fitted to the Michaelis-Menten equation and determination of the *K*_m_ and *V*_max_ values of purified ASNase by Lineweaver-Burk plot (**c**).

The enzyme was active over broad pH range, with its activity gradually increasing up to pH 7.0 (Fig. 11a). The enzymatic activity decreased at other than optimal pH values, with up to 31.63 and 18.47% of the maximal activity retained at pH values of 9.0 and 5.0, respectively. The purified enzyme was stable at a pH range of 7.0 to 8.5, losing only 7.5 and 26.60% of its original activity at pH values of 9.0 and 10.0. The maximal activity of the purified ASNase was recorded at 37 °C, with the enzyme activity gradually decreasing at temperatures above this value (Fig. 11b). The lowest activity of the ASNase was observed at 80 °C, where an activity of 13.90 U/mL was measured. The ASNase was exhibiting thermostability over a temperature range of 30–70 °C. The parameters *K*_m_ and *V*_max_ were determined for the purified ASNase through a steady-state kinetic analysis (Fig. 11c). The plot of the reaction velocity versus substrate concentration exhibited a typical hyperbolic saturation curve that was fitted to the Michaelis-Menten equation, yielding the kinetics constant. The Lineweaver Burk plot showed that the *K*_m_ and *V*_max_ values of enzyme using L-asparagine as substrate were 0.049995 M and 45.45 μmol/ml/min, respectively.

### Anticancer activity of ASNase against cancer cell lines

**Figure 12:**
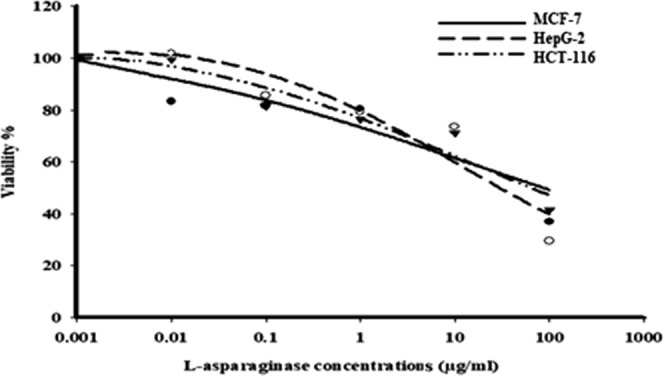
Dose response curves of *B. licheniformis* against MCF-7, HepG-2 and HCT-116 cancer cells.

The viability (%) of MCF-7, HepG-2 and HCT-116 cell lines was used as an indicator of cell toxicity after cells were treated with different concentrations of ASNase produced by *B. licheniformis* for 72 h. The resulting toxicity of the enzyme toward MCF-7, HepG-2 and HCT-116 cells was dose dependent since gradual increases in the dose of the ASNase enzyme resulted in a gradual inhibition of cell growth (Fig. 12). The purified ASNase enzyme was shown to selectively inhibited cancer cell reproduction without any cytotoxic effect toward the non-carcinogenic human cell line (normal cells) at the concentrations studied. Morphological changes in the cancer cell lines after treatment with bacterial ASNase were evaluated under an inverted microscope. The cells showed signs of detachment from the surface of the wells, which denoted cell death. The apoptotic cells also showed specific characteristics, such as cellular rounding, shrinkage, membrane blabbing and loss of cell adhesion. The cytotoxic effect of the *B. licheniformis* ASNase showed significant toxic activity toward HepG-2 cells (IC_50_ value of 11.66 µg/mL), which was more than the effect observed against MCF-7 and HCT-116 cells (IC_50_ values of 14.55 and 17.0226 µg/mL, respectively).

## Discussion

The current commercially available ASNases produced by bacteria can lead to hypersensitivity and toxicity during therapy, demonstrating the need to identify new sources of these enzymes^24^. In the present study, 40 marine bacterial isolates were obtained from the Red Sea coast in the southern region of Saudi Arabia using a Millipore filter method while the serial dilution method was unfavorable in view of the difficulty of isolating marine bacteria since most them uncultured. The marine environment of the Red Sea is a potent source of bacterial species that may be useful in the discovery of novel bioactive compounds^7,25^. In our study, 28 marine bacterial isolates showed the ability to produce ASNase to different extents. Marine bacteria are believed to be a promising source of anticancer ASNases through the use of submerged fermentation, which has unique characteristics^26^. Additional screening was conducted to assess ASNase production using freely suspended and immobilized cell techniques. The production of enzyme by immobilized bacterial cells was greater than that observed by the freely suspended cells, with an approximately 43.9%. Kattimani *et al*.^27^ reported that enzyme production of immobilized *Streptomyces gulbargensis* cells was enhanced by approximately 30% compared to conventional free cell fermentation. The grater ASNase production by immobilized cells could be due to the increased growth of cells in the alginate matrix, as well as the enzyme activities being maintained at higher levels than those of the free cells^28^. Cell immobilization is considered a promising approach for enhancing the production of ASNase, as this approach offers numerous benefits, including a sustained process over a long period of time, the re-use of immobilized beads, easy harvesting of products, reactor productivity, higher catalytic efficiency and lowered processing costs^7^.

The SEM photographed of the free and immobilized bacterial cells in calcium alginate capsules indicates the purity of the bacterial strain and the proper immobilization of the bacterial cells inside the polymeric capsules, in addition to the predicted prolonged shelf life of the dried immobilized capsules which have the ability to allow the bacterial cells to restore their activity after exposure to various conditions^29^.

Molecular identification of the bacterial 16S rRNA gene and phylogenetic analysis confirmed the classification of the selected isolate, KKU-KH14 as *B. licheniformis*, which belongs to the phylum Firmicutes. The predominant bacterial phyla in the Red Sea are Proteobacteria, Firmicutes, Fusobacteria, Bacteroidetes and Spirochetes^30^. ASNases have been identified in several marine bacteria while, the most abundant bacterial isolates were *Bacillus* sp. and *Pseudomonas* sp^31^. The abundance of *Bacillus* sp. throughout marine habitats is associated with its ability to produce spores, which exhibit high resilience to environmental stresses^32^.

The optimization conditions of ASNase production by the *B. licheniformis* identified in this study were in agreement with the results of a study by Kumar *et al*.^33^, who reported that ASNase production is strongly influenced by the composition of fermentation media and the culture conditions, including time, temperature, pH and agitation rate. The maximal production of the ASNase investigated in this study was observed at 72 hours, in the stationary phase of bacterial growth, indicating that enzyme production and bacterial growth were linked. The culturing period plays an essential role in ASNase productivity by bacterial strains, with optimal production observed for *S. marcescens* and *B. methylotrophicus* at 96 hours^34^, for *Bacillus* sp. R36 at 24 hours^35^, for *Lactobacillus salivarius* at 120 hours^26^ and for *Bacillus subtilis* at 36 hours^36^.

The pH of a medium significantly impacts numerous enzyme processes and the movement of different components across the microbial cell membrane^37^. The most suitable pH for ASNase production is dependent on the specific strain, differences in which could be due to fermentation conditions and the specific genetic characteristics of a microbial species^38^. In this study, the maximum ASNase production occurred at pH 6.5, a result that is similar to that observed by Pradhan *et al*.^36^, who reported that ASNase production by *Bacillus subtilis* occurred optimally at 6.5 pH. Moreover, Mahajan *et al*.^39^ reported that ASNase production by *B. licheniformis* occurs over a pH range of 5.5–7.5, with maximum enzyme production occurring at pH 6.0.

Maximum enzyme productivity of the ASNase evaluated in this study was obtained at 37 °C, similar to the ASNase from *Bacillus subtilis*^36^, whereas the optimal temperature for the production of enzyme by *Streptomyces olivaceus* was 35 °C^38^. In contrast, the optimal production of ASNases from *Bacillus* PG02 and *Bacillus* PG04 occurred at pH 6–7.5, which is close to physiological pH^40^. This feature is a major requirement for the antitumor activity of ASNases with optimal temperatures of 37 °C.

The agitation process is essential for optimal bacterial growth and ASNase production^38^. The optimal agitation rate for enzyme production was observed at 250 rpm. Because microorganisms growing in a submerged culture use oxygen dissolved in the medium, this is critical for the growth requirements of the organism and biosynthesis of specific end products. Maysa *et al*.^35^ revealed that oxidation-reduction mechanisms in the fermentation mixture may exert a chemical influence on the biosynthetic of ASNase productivity. The best agitation rate for ASNase production was determined to be 200 rpm for *Streptomyces olivaceus*^38^, 150 rpm for *Bacillus subtilis*^36^ and 250 rpm for *Lactobacillus salivarius*^26^.

The nutritional requirements for maximal ASNase synthesis varies among microorganisms, and the rate of synthesis varies in the same organism as a function of culture conditions^6^. Different carbon sources in media formulations were used to enhance growth and the synthesis of primary metabolites, such as enzymes. The ASNase production in cells of *B. licheniformis* was observed to depend on specific factors. Glucose was the best carbon source for the bacterial growth and ASNase production, similar to the results reported for *Bacillus* sp.^41^. In contrast, Wakil and Adelegan^42^ showed that the optimal carbon source was mannitol for ASNase production in *Bacillus circulans*, *Streptococcus* sp. and *Bacillus polymyxa*; maltose for *Streptococcus* sp. and *Bacillus firmus*; and sucrose for *Paenibacillus validus*, which also aided in stabilizing the enzyme.

Organic and inorganic nitrogen sources are microbial metabolized to produce amino acids, nucleic acids, proteins, and cell wall components to enhance the production of ASNase. The optimum nitrogen source for ASNase production in this study was (NH_4_)_2_SO_4_. Hosamani and Kaliwal^43^ reported that ammonium sulfate was the optimum nitrogen source for ASNase production in *B. licheniformis*, while Baskar and Renganathan^44^ reported that ammonium chloride was more favorable. In a previous study, the nitrogen source was the second most crucial factor affecting ASNase production and was also important for the growth of organisms^42^. The authors showed that the ideal nitrogen source for ASNase production by *Bacillus circulans* and *Streptococcus* sp. was casein, yeast extract for *Bacillus polymyxa* and *Bacillus firmus*, and NaNO_3_ for *Streptococcus* sp. and *Paenibacillus validus*. Yeast extract and casein were shown to promote cell growth and ASNase production, while increased concentrations of these components resulted in decreased ASNase synthesis, which may be due to the presence of a high substrate concentration and the induction of proteolytic enzyme^42^.

An ASNases was purified by ammonium sulfate precipitation, dialysis and gel filtration chromatography using Sephadex G-100 chromatography^22^. The precipitation ASNases via ammonium is the most widely used method to purify these enzymes, followed by dialysis to remove excess salt. Furthermore, complete purification of ASNases has been achieved using chromatographic techniques^45^. Specific activity (units/mg) is a measure of enzyme purity, the value of which increases as the purity of an enzyme increases since the amount of contaminating protein typically decreases. The results of this study agreed with the findings of Farag *et al*.^7^, where a *Streptomyces fradiae* NEAE-82 ASNase was purified and exhibited a final specific activity of 30.636 U/mg protein and was purified 3.33-fold^29^.

An SDS-PAGE analysis of the purified ASNase showed a single distinct protein band with a molecular weight of 37 kDa (Fig. 12). These results were in accordance with other reports^46,47^ for ASNases from *B. licheniformis*. The molecular weights of ASNases varies according to the microbial source of enzyme. The molecular weight of an ASNase from *Streptomyces fradiae* NEAE-82 was determined to be approximately 53 kDa^29^. In contrast, SDS-PAGE analysis of a purified ASNase from *Pseudonocardia endophytic* VUK-10 revealed a distinct single peptide chain with molecular weight of 120 kDa^22^. A purified *Vibrio cholerae* ASNase that was recombinant overexpressed in *E. coli* exhibited a molecular weight of approximately 36.6 kDa^1^. The results of SDS-PAGE and glutaminase activity assay for the purified ASNase in this study indicated that the enzyme was pure and free of glutaminase activity. These results were similar to those observed in numerous previous reports that described *B. licheniformis* as producing ASNases with low or no glutaminase activity^39,46,47^. The concern associated with the commercial use of ASNases is its high glutaminase activity, which can cause liver dysfunction, pancreatitis, leucopenia, neurological seizures, and coagulation abnormalities that lead to intracranial thrombosis^13,14,46^.

The optimal pH and temperature at which the optimal activity and stability of the ASNase evaluated in this study, showed that the enzyme could function well in the human body. Enzymes that are stable at physiological temperature and pH is a desirable characteristic for proteins that are used as therapeutics. Our results were similar to those of Ghasemi *et al*.,^7^ who observed that the optimum temperature for the activity of an ASNase from the marine bacterium *Halomonas elongata* was 37 °C, while the enzyme showed maximum activity over a wide pH range (6–9) that was similar to values observed in the human body. An ASNase from *B. licheniformis* was maximally active over a pH range of 6.0 to 10.0 at a temperature of 40 °C, with maximal enzyme stability observed at pH 9.0^46^. In addition, El-Naggar *et al*.^29^ reported that ASNase are amidases that are generally active and stable at neutral and alkaline pH. They observed that the optimum temperature for the activity and stability of an ASNase from *Streptomyces fradiae* NEAE-82 occurred at 40 and 50 °C, respectively. In addition, the ideal temperature for the activity of a *B. licheniformis* ASNase occurred at 37 °C. The observed increase in the thermostability of this enzyme may be explained by changes in the charges of specific amino acids, which can destabilize the proteins^47^.

In this study, the kinetic constants *K*_m_ and *V*_max_ of the purified ASNase were determined by steady-state kinetic analysis fitted to the Michaelis-Menten equation. The *K*_m_ reflects the affinity of the enzyme for its substrate while *V*_max_ is a measure of substrate turnover and is expressed in units of product formed per unit of time^22^. The Lineweaver Burk plot results showed a high affinity of the enzyme toward the substrate, with a measured *K*_m_ value of 0.049995 M and a *V*_max_ value of 45.45 μmol/ml/min. Many factors affect the kinetic parameters of enzymes, such as the source, type, and form of an enzyme, as well as changes in enzyme conditions and assay procedures^7^. Sudhir *et al*.^47^ reported *K*_m_ and *V*_max_ values for an ASNase from *B. licheniformis* of 0.42 mM and 2778.9 μmol min, respectively, while *K*_m_ and *V*_max_ values of the enzyme purified from the same bacterium were measured as 1.4 × 10^5^ M and 4.03 IU, respectively^46^. In addition, the *K*_m_ and *V*_max_ values for an ASNase from *Streptomyces fradiae* NEAE-82 were 0.01007 M and 95.08 Uml^−1^min^−1^, respectively^29^.

Cancer rates are rising worldwide, and breast cancer is considered to be the second leading cause tumor-related deaths in women in Saudi Arabia^48^, with colon cancer being the third leading cause of death, and a high mortality rate observed for breast cancer worldwide^49^. Liver malignancy is another common diagnosis in middle-eastern patients who suffer from chronic liver disease^50^. The toxicity of the ASNase produced by *B. licheniformis* in this study was evaluated for cancer cell lines, the results of which showed that the enzyme was highly toxic toward HepG-2 cells, more so than against MCF-7 and HCT-116 cells. These findings were comparable to those of other studies^46,51^, where ASNases from marine *Bacillus* sp. and *B. licheniformis* strains exhibited the highest cytotoxic effect against the cancer cell lines Jurkat clone E6-1, MCF-7 and K-562, with IC_50_ values of 0.22, 0.78 and 0.153 IU, respectively. However, the results of the current study were superior to those described previously^7^ for *Halomonas elongate*, where an ASNase inhibited the growth of human leukemia cell lines with IC_50_ values of 1–2 U/mL. Furthermore, an ASNase from *Bacillus* sp. R36 inhibited the growth of two human cell lines (HCT-116 and HepG-2), with IC_50_ values of 218.and 112.19 μg/mL, respectively^35^. In addition, Shafei *et al*.^52^ described an ASNase that inhibited the growth of three human cell lines, with values of IC_50_ values of 12.5, and 37 μg/mL against breast, hepatocellular and prostate carcinoma, respectively. ASNases have been shown to inhibit the glycosylation of several forms of newly synthesized proteins^53^. El-Naggar *et al*.^29^ suggested that ASNases can alter the interactions between the microvasculature of endothelial cells, colon cancer cells, and extracellular matrix components, resulting in colon cancer cell disruption. After being converted into oxaloacetic acid, asparagine is involved in the Krebs cycle, influencing cell metabolism^54^. In addition, intracellular asparagine contributes to the uptake of extracellular serine, which is utilized to synthesize nucleic acids^55^. Since the multiplication of the human leukemic cell lines and other tumor cells requires excessive amounts of these amino acids to produce sufficient energy and synthesize biomolecules, decreases in the supply of asparagine are likely to prevent the growth of these cells^56^. The microscopic observations made in this study further confirmed our results. Apoptotic cells exhibit condensed nuclear heterochromatin and partitioning of the cytoplasm and nucleus into membrane-bound vesicles^25,57^. In addition, Karpel-Massler *et al*.^2^ observed that cancer cell lines are susceptible to the repression of proliferation by ASNase, which stimulates apoptosis by decreasing mitochondrial membrane potential. Muñoz-Pinedo^58^ elucidated the action of anticancer enzymes that catalyze the conversion of specific amino acids into a form that is unavailable to cells, leading to starvation conditions. In addition, Ueno *et al*.^59^ showed that tumor cells do not have an L-asparagine synthetase gene and cannot produce L-asparagine such that they will starve and cause apoptosis to occur, and the absence of asparagine disrupts the cell cycle.

## Conclusion

The results of this study demonstrate the potential of the marine environment of the Red Sea as a potent source of bacterial strains for the discovery of anticancer drugs. The identification of strains with improved ASNase yields has attracted a great deal of attention to improve the cost-effectiveness of these enzymes. Efficient production of ASNase was achieved through Ca-alginate immobilization of *B. licheniformis* cells. The rate of ASNase synthesis depends on complex interactions among pH, temperature, agitation and the presence of specific nutrients. The properties, kinetic parameters, and anticancer activity of the purified enzyme demonstrated its excellent potential for use as a potent anticancer agent. However, more detailed studies are needed to evaluate this possibility, including *in vivo* immunogenicity and half-life determination assays, pharmacokinetic and pharmacodynamic profiling in model animals, and human clinical trials.

## Data Availability

The datasets generated during this study are available from the corresponding author upon reasonable request. The data that support the findings of this study are available from GenBank and the KKU microbial collection.
